# Bis[bis­(3,5-dimethyl-1*H*-pyrazol-1-yl)­borato]cobalt(II)

**DOI:** 10.1107/S1600536811020976

**Published:** 2011-06-11

**Authors:** Moayad Hossaini Sadr, Behzad Soltani, James T. Engle, Christopher J. Ziegler, M. Taheri

**Affiliations:** a Department of Chemistry, Azarbaijan University of Tarbiat Moallem, Tabriz, Iran; b Department of Chemistry, University of Akron, Akron, OH, USA

## Abstract

The asymmetric unit of the title compound, [Co(C_10_H_16_BN_4_)_2_], comprises one unit of the complex. The geometry around the Co^II^ ion is a distorted tetra­hedron. The dihedral angles between the pyrazole rings in the two ligands are 47.19 (15) and 47.20 (16)°, while that between the coordination planes is 79.77 (7)°.

## Related literature

For standard values of bond lengths, see: Allen *et al.* (1987 ▶). For background on pyrazolates and their complexes, see, for example; Trofimenko (1967 ▶); Trofimenko (1999 ▶); Trofimenko (2004 ▶); Sadr *et al.* (2008 ▶); Ruman *et al.* (2003 ▶); Krzystek *et al.* (2010 ▶).
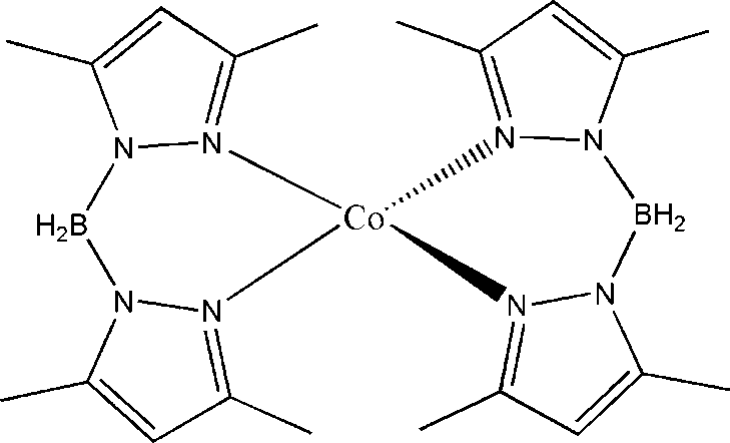


## Experimental

### 

#### Crystal data

[Co(C_10_H_16_BN_4_)_2_]
*M* *_r_* = 465.09
Monoclinic, 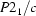

*a* = 8.351 (5) Å
*b* = 14.012 (9) Å
*c* = 19.833 (13) Å
β = 93.281 (7)°
*V* = 2317 (3) Å^3^
*Z* = 4
Mo *K*α radiation
μ = 0.77 mm^−1^
*T* = 100 K
0.25 × 0.15 × 0.12 mm

#### Data collection

Bruker APEXII CCD diffractometer
Absorption correction: multi-scan (*SADABS*; Sheldrick, 2005 ▶) *T*_min_ = 0.832, *T*_max_ = 0.914
18648 measured reflections
5048 independent reflections
3947 reflections with *I* > 2σ(*I*)
*R*_int_ = 0.046

#### Refinement

*R*[*F* ^2^ > 2σ(*F* ^2^)] = 0.050
*wR*(*F* ^2^) = 0.122
*S* = 1.04
5048 reflections
288 parameters
H-atom parameters constrained
Δρ_max_ = 0.60 e Å^−3^
Δρ_min_ = −0.61 e Å^−3^

### 

Data collection: *APEX2* (Bruker, 2005 ▶); cell refinement: *SAINT* (Bruker, 2005 ▶); data reduction: *SAINT*; program(s) used to solve structure: *SHELXS97* (Sheldrick, 2008 ▶); program(s) used to refine structure: *SHELXL97* (Sheldrick, 2008 ▶); molecular graphics: *SHELXTL* (Sheldrick, 2008 ▶); software used to prepare material for publication: *SHELXTL* and *PLATON* (Spek, 2009 ▶).

## Supplementary Material

Crystal structure: contains datablock(s) global, I. DOI: 10.1107/S1600536811020976/bg2405sup1.cif

Structure factors: contains datablock(s) I. DOI: 10.1107/S1600536811020976/bg2405Isup2.hkl

Additional supplementary materials:  crystallographic information; 3D view; checkCIF report

## Figures and Tables

**Fig. 1.**
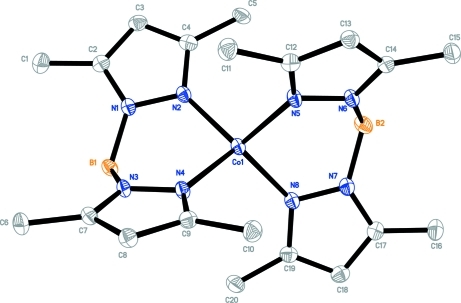
The ORTEP plot of the title compound, showing 40% probability displacement ellipsoids and the atomic numbering. The H atoms were omitted for clarity.

## supplementary crystallographic information

### Comment

During the last 40 years anionic polypyrazolylborate ligands (or Trofimeko's scorpionates) have proven to be popular and versatile ligands, binding to a wide variety of transition-metal ions (Trofimenko, 1967; Trofimenko, 1999; Trofimenko, 2004). In addition to variation of the coordinated metal ion or replacing H atoms bonded to a B centre by alkyl moieties, variation of type and number of substituents on the pyrazolyl rings allows for the synthesis of a large number of scorpionate complexes (Ruman *et al.*, 2003; Krzystek *et al.*, 2010). Cobalt complexes with bis pyrazolylborate ligands are of inherent interest in terms of understanding the electronic structure of Co^II^ as a function of coordination environment, even though, Co^II^ complexes themselves are of specific, biological interest as models for the active sites of many metalloproteins. In continuation of our work on the synthesis and structure of pyrazolate complexes (Sadr *et al.*, 2008), we determined the crystal structure of the title compound, [Co(C_10_ H_16_ B N_4_)_2_] (1).

The asymmetric unit of (1) (Fig. 1), comprises one unit of the complex. The bond lengths and angles are within the normal ranges (Allen, *et al.*, 1987). The geometry around Co^II^ is a distorted tetrahedron defined by N1, N4, N5, and N8 in the coordinated pyrazolate ligands. (Co-N, N-Co-N' ranges: 1.990 (2)-1.996 (2)Å and 97.03 (9)-137.03 (10)°, respectively ). The dihedral angle between the coordination planes (N1–Co1–N4 and N5–Co1–N8) is 79.77 (7)°, and the ones between the pyrazolate rings in each ligand are 47.19 (15) and 47.20 (16)°.

The structure does not present any kind of H-bonding interactions.

### Experimental

Tetrahydrofurane (25 ml) solution of potassium pyrazolborate (2 mmol, 0.48 g) was added into the stock water solution of Co(NO_3_)_2_,6H_2_O (50 ml, 0.1 *M*). The reaction mixture was stirred for 3 h and the resulting product was extracted with CH_2_Cl_2_ (50 ml). The organic phase was washed twice with water (100 ml) and CH2Cl2 was removed on a rotary evaporator. The solid residue was dissolved in THF and left for crystallization from THF/*n*-Hexane (3/1) mixture at ambient temperature by slow evaporation. After 3 days, block purple crystals were collected. Single crystals suitable for *X*-ray diffraction analysis were obtained after 4 days.

### Refinement

All hydrogen atoms were positioned geometrically with C–H = 0.95–0.98 Åand included in a riding model approximation with *U*_iso_ (H) = 1.2 or 1.5 *U*_eq_ (C), except the B-bound H atoms which were located from the difference Fourier map and constrained to refine with the parent atom with *U*_iso_ (H) = 1.2 *U*_eq_ (B). A rotating model were applied to the methyl groups.

### Crystal data

**Table:**

[Co(C_10_H_16_BN_4_)_2_]	*F*(000) = 980
*M**_r_* = 465.09	*D*_x_ = 1.333 Mg m^−^^3^
Monoclinic, *P*2_1_/*c*	Mo *K*α radiation, λ = 0.71073 Å
Hall symbol: -P 2ybc	Cell parameters from 5094 reflections
*a* = 8.351 (5) Å	θ = 2.5–28.1°
*b* = 14.012 (9) Å	µ = 0.77 mm^−^^1^
*c* = 19.833 (13) Å	*T* = 100 K
β = 93.281 (7)°	Block, purple
*V* = 2317 (3) Å^3^	0.25 × 0.15 × 0.12 mm
*Z* = 4	

### Data collection

**Table:**

Bruker APEXII CCD diffractometer	5048 independent reflections
Radiation source: fine-focus sealed tube	3947 reflections with *I* > 2σ(*I*)
graphite	*R*_int_ = 0.046
φ and ω scans	θ_max_ = 27.0°, θ_min_ = 1.8°
Absorption correction: multi-scan (*SADABS*; Sheldrick, 2008)	*h* = −10→10
*T*_min_ = 0.832, *T*_max_ = 0.914	*k* = −17→17
18648 measured reflections	*l* = −25→24

### Refinement

**Table:**

Refinement on *F*^2^	Primary atom site location: structure-invariant direct methods
Least-squares matrix: full	Secondary atom site location: difference Fourier map
*R*[*F*^2^ > 2σ(*F*^2^)] = 0.050	Hydrogen site location: inferred from neighbouring sites
*wR*(*F*^2^) = 0.122	H-atom parameters constrained
*S* = 1.04	*w* = 1/[σ^2^(*F*_o_^2^) + (0.0516*P*)^2^ + 2.3676*P*] where *P* = (*F*_o_^2^ + 2*F*_c_^2^)/3
5048 reflections	(Δ/σ)_max_ < 0.001
288 parameters	Δρ_max_ = 0.60 e Å^−^^3^
0 restraints	Δρ_min_ = −0.61 e Å^−^^3^

### Special details

**Table:**

Experimental. 'Ratio of minimum to maximum apparent transmission: 0.1661'
Geometry. All e.s.d.'s (except the e.s.d. in the dihedral angle between two l.s. planes) are estimated using the full covariance matrix. The cell e.s.d.'s are taken into account individually in the estimation of e.s.d.'s in distances, angles and torsion angles; correlations between e.s.d.'s in cell parameters are only used when they are defined by crystal symmetry. An approximate (isotropic) treatment of cell e.s.d.'s is used for estimating e.s.d.'s involving l.s. planes.
Refinement. Refinement of *F*^2^ against ALL reflections. The weighted *R*-factor *wR* and goodness of fit *S* are based on *F*^2^, conventional *R*-factors *R* are based on *F*, with *F* set to zero for negative *F*^2^. The threshold expression of *F*^2^ > σ(*F*^2^) is used only for calculating *R*-factors(gt) *etc*. and is not relevant to the choice of reflections for refinement. *R*-factors based on *F*^2^ are statistically about twice as large as those based on *F*, and *R*- factors based on ALL data will be even larger.

### Fractional atomic coordinates and isotropic or equivalent isotropic displacement parameters (Å^2^)

**Table:**

	*x*	*y*	*z*	*U*_iso_*/*U*_eq_	
Co1	0.10933 (4)	0.19693 (2)	0.197855 (17)	0.01815 (12)	
N1	0.1543 (3)	0.15049 (15)	0.34192 (11)	0.0192 (5)	
N2	0.0445 (3)	0.15101 (15)	0.28722 (11)	0.0193 (5)	
N3	0.3688 (2)	0.25721 (15)	0.29484 (10)	0.0172 (5)	
N4	0.2967 (3)	0.27492 (16)	0.23208 (11)	0.0192 (5)	
N5	−0.0682 (3)	0.28065 (15)	0.16145 (11)	0.0194 (5)	
N6	−0.1491 (3)	0.25825 (16)	0.10128 (11)	0.0196 (5)	
N7	0.0520 (3)	0.14005 (16)	0.05685 (11)	0.0211 (5)	
N8	0.1649 (3)	0.13899 (16)	0.11056 (11)	0.0201 (5)	
C1	0.1616 (4)	0.1256 (2)	0.46705 (14)	0.0293 (7)	
H1A	0.2306	0.0690	0.4695	0.044*	
H1B	0.0816	0.1208	0.5012	0.044*	
H1C	0.2270	0.1829	0.4755	0.044*	
C2	0.0784 (3)	0.13186 (19)	0.39839 (14)	0.0219 (6)	
C3	−0.0833 (3)	0.1208 (2)	0.38042 (14)	0.0242 (6)	
H3	−0.1662	0.1071	0.4098	0.029*	
C4	−0.1002 (3)	0.13375 (19)	0.31117 (14)	0.0228 (6)	
C5	−0.2482 (3)	0.1303 (2)	0.26574 (15)	0.0274 (6)	
H5A	−0.2726	0.1944	0.2483	0.041*	
H5B	−0.3379	0.1072	0.2910	0.041*	
H5C	−0.2315	0.0870	0.2280	0.041*	
C6	0.5782 (3)	0.3276 (2)	0.37451 (14)	0.0246 (6)	
H6A	0.5128	0.3126	0.4126	0.037*	
H6B	0.6262	0.3910	0.3811	0.037*	
H6C	0.6634	0.2798	0.3718	0.037*	
C7	0.4748 (3)	0.32668 (18)	0.31042 (13)	0.0191 (5)	
C8	0.4721 (3)	0.39084 (19)	0.25731 (13)	0.0206 (6)	
H8	0.5346	0.4471	0.2543	0.025*	
C9	0.3593 (3)	0.35616 (18)	0.20939 (13)	0.0195 (5)	
C10	0.3077 (3)	0.3951 (2)	0.14138 (14)	0.0251 (6)	
H10A	0.3101	0.3441	0.1077	0.038*	
H10B	0.3808	0.4464	0.1295	0.038*	
H10C	0.1985	0.4204	0.1424	0.038*	
C11	−0.0646 (3)	0.4077 (2)	0.24764 (14)	0.0261 (6)	
H11A	−0.0821	0.3625	0.2842	0.039*	
H11B	−0.1234	0.4669	0.2554	0.039*	
H11C	0.0502	0.4217	0.2465	0.039*	
C12	−0.1234 (3)	0.36511 (18)	0.18175 (13)	0.0204 (6)	
C13	−0.2387 (3)	0.39813 (19)	0.13439 (13)	0.0210 (6)	
H13	−0.2965	0.4564	0.1357	0.025*	
C14	−0.2524 (3)	0.32872 (19)	0.08469 (13)	0.0201 (5)	
C15	−0.3648 (3)	0.3250 (2)	0.02312 (14)	0.0260 (6)	
H15A	−0.3068	0.3027	−0.0155	0.039*	
H15B	−0.4081	0.3889	0.0135	0.039*	
H15C	−0.4529	0.2810	0.0310	0.039*	
C16	0.0339 (4)	0.1101 (2)	−0.06737 (14)	0.0282 (6)	
H16A	−0.0476	0.0599	−0.0666	0.042*	
H16B	0.1094	0.0952	−0.1019	0.042*	
H16C	−0.0181	0.1715	−0.0779	0.042*	
C17	0.1225 (3)	0.11597 (19)	0.00018 (14)	0.0225 (6)	
C18	0.2834 (3)	0.09960 (19)	0.01625 (14)	0.0248 (6)	
H18	0.3625	0.0813	−0.0138	0.030*	
C19	0.3061 (3)	0.11533 (19)	0.08547 (14)	0.0221 (6)	
C20	0.4570 (3)	0.1113 (2)	0.12963 (15)	0.0282 (6)	
H20A	0.5029	0.1755	0.1342	0.042*	
H20B	0.5340	0.0688	0.1093	0.042*	
H20C	0.4329	0.0871	0.1743	0.042*	
B1	0.3357 (3)	0.1598 (2)	0.32884 (16)	0.0201 (6)	
H1D	0.3987	0.1553	0.3713	0.024*	
H1E	0.3674	0.1081	0.2999	0.024*	
B2	−0.1278 (4)	0.1565 (2)	0.07122 (17)	0.0237 (7)	
H2A	−0.1621	0.1089	0.1028	0.028*	
H2B	−0.1937	0.1503	0.0296	0.028*	

### Atomic displacement parameters (Å^2^)

**Table:**

	*U*^11^	*U*^22^	*U*^33^	*U*^12^	*U*^13^	*U*^23^
Co1	0.01670 (19)	0.01856 (19)	0.01875 (19)	−0.00036 (14)	−0.00268 (13)	−0.00079 (15)
N1	0.0196 (12)	0.0180 (11)	0.0196 (11)	0.0006 (9)	−0.0028 (9)	−0.0001 (9)
N2	0.0178 (11)	0.0188 (11)	0.0208 (11)	−0.0011 (9)	−0.0026 (9)	−0.0018 (9)
N3	0.0166 (11)	0.0174 (11)	0.0171 (11)	−0.0004 (8)	−0.0039 (9)	0.0019 (8)
N4	0.0178 (11)	0.0210 (11)	0.0183 (11)	−0.0002 (9)	−0.0035 (9)	0.0024 (9)
N5	0.0185 (11)	0.0190 (11)	0.0200 (11)	−0.0002 (9)	−0.0052 (9)	−0.0024 (9)
N6	0.0178 (12)	0.0201 (11)	0.0201 (11)	0.0012 (9)	−0.0040 (9)	−0.0023 (9)
N7	0.0217 (12)	0.0207 (12)	0.0203 (12)	0.0013 (9)	−0.0026 (9)	−0.0016 (9)
N8	0.0189 (11)	0.0208 (12)	0.0203 (11)	0.0003 (9)	−0.0026 (9)	−0.0009 (9)
C1	0.0339 (17)	0.0305 (16)	0.0232 (15)	−0.0030 (13)	−0.0002 (12)	0.0009 (12)
C2	0.0250 (14)	0.0173 (13)	0.0236 (14)	−0.0013 (11)	0.0034 (11)	−0.0012 (11)
C3	0.0229 (14)	0.0239 (14)	0.0265 (15)	−0.0034 (11)	0.0063 (11)	−0.0025 (12)
C4	0.0205 (14)	0.0184 (13)	0.0296 (15)	−0.0008 (11)	0.0030 (11)	−0.0027 (11)
C5	0.0161 (14)	0.0346 (16)	0.0315 (16)	−0.0024 (12)	0.0001 (11)	−0.0056 (13)
C6	0.0260 (15)	0.0248 (15)	0.0223 (14)	−0.0041 (11)	−0.0053 (11)	−0.0017 (11)
C7	0.0171 (13)	0.0185 (13)	0.0213 (13)	0.0010 (10)	−0.0016 (10)	−0.0013 (10)
C8	0.0209 (14)	0.0179 (13)	0.0229 (14)	−0.0027 (10)	0.0007 (11)	0.0008 (11)
C9	0.0196 (13)	0.0185 (13)	0.0205 (13)	0.0022 (10)	0.0019 (10)	0.0018 (10)
C10	0.0267 (15)	0.0263 (15)	0.0222 (14)	−0.0008 (11)	−0.0004 (11)	0.0046 (11)
C11	0.0287 (16)	0.0251 (15)	0.0241 (15)	−0.0003 (12)	−0.0012 (12)	−0.0043 (11)
C12	0.0208 (14)	0.0174 (13)	0.0230 (14)	−0.0015 (10)	0.0024 (11)	−0.0003 (10)
C13	0.0206 (14)	0.0195 (13)	0.0231 (14)	0.0029 (10)	0.0019 (11)	0.0022 (11)
C14	0.0186 (13)	0.0230 (14)	0.0187 (13)	0.0005 (10)	0.0020 (10)	0.0016 (10)
C15	0.0254 (15)	0.0299 (16)	0.0223 (14)	0.0035 (12)	−0.0038 (11)	−0.0006 (12)
C16	0.0355 (17)	0.0268 (15)	0.0218 (15)	0.0062 (13)	−0.0035 (12)	−0.0017 (12)
C17	0.0278 (15)	0.0172 (13)	0.0224 (14)	0.0013 (11)	0.0009 (11)	−0.0008 (11)
C18	0.0269 (15)	0.0218 (14)	0.0261 (15)	0.0035 (11)	0.0056 (11)	0.0017 (11)
C19	0.0212 (14)	0.0187 (13)	0.0265 (15)	0.0025 (10)	0.0028 (11)	0.0028 (11)
C20	0.0207 (15)	0.0330 (16)	0.0308 (16)	0.0017 (12)	−0.0001 (12)	0.0024 (13)
B1	0.0171 (15)	0.0193 (15)	0.0233 (15)	−0.0001 (11)	−0.0021 (11)	0.0039 (12)
B2	0.0194 (16)	0.0215 (15)	0.0296 (17)	−0.0016 (12)	−0.0029 (12)	−0.0053 (13)

### Geometric parameters (Å, °)

**Table:**

Co1—N2	1.990 (2)	C6—H6C	0.9800
Co1—N8	1.991 (2)	C7—C8	1.384 (4)
Co1—N5	1.993 (2)	C8—C9	1.387 (4)
Co1—N4	1.996 (2)	C8—H8	0.9500
N1—C2	1.344 (3)	C9—C10	1.495 (4)
N1—N2	1.380 (3)	C10—H10A	0.9800
N1—B1	1.557 (4)	C10—H10B	0.9800
N2—C4	1.345 (3)	C10—H10C	0.9800
N3—C7	1.340 (3)	C11—C12	1.494 (4)
N3—N4	1.374 (3)	C11—H11A	0.9800
N3—B1	1.554 (4)	C11—H11B	0.9800
N4—C9	1.341 (3)	C11—H11C	0.9800
N5—C12	1.340 (3)	C12—C13	1.385 (4)
N5—N6	1.374 (3)	C13—C14	1.385 (4)
N6—C14	1.340 (3)	C13—H13	0.9500
N6—B2	1.560 (4)	C14—C15	1.498 (4)
N7—C17	1.341 (3)	C15—H15A	0.9800
N7—N8	1.381 (3)	C15—H15B	0.9800
N7—B2	1.561 (4)	C15—H15C	0.9800
N8—C19	1.347 (3)	C16—C17	1.495 (4)
C1—C2	1.496 (4)	C16—H16A	0.9800
C1—H1A	0.9800	C16—H16B	0.9800
C1—H1B	0.9800	C16—H16C	0.9800
C1—H1C	0.9800	C17—C18	1.382 (4)
C2—C3	1.385 (4)	C18—C19	1.393 (4)
C3—C4	1.385 (4)	C18—H18	0.9500
C3—H3	0.9500	C19—C20	1.494 (4)
C4—C5	1.488 (4)	C20—H20A	0.9800
C5—H5A	0.9800	C20—H20B	0.9800
C5—H5B	0.9800	C20—H20C	0.9800
C5—H5C	0.9800	B1—H1D	0.9691
C6—C7	1.495 (4)	B1—H1E	0.9704
C6—H6A	0.9800	B2—H2A	0.9695
C6—H6B	0.9800	B2—H2B	0.9693
			
N2—Co1—N8	137.03 (10)	N4—C9—C8	109.3 (2)
N2—Co1—N5	106.14 (10)	N4—C9—C10	121.0 (2)
N8—Co1—N5	97.50 (10)	C8—C9—C10	129.8 (2)
N2—Co1—N4	97.03 (9)	C9—C10—H10A	109.5
N8—Co1—N4	107.57 (10)	C9—C10—H10B	109.5
N5—Co1—N4	110.71 (10)	H10A—C10—H10B	109.5
C2—N1—N2	109.4 (2)	C9—C10—H10C	109.5
C2—N1—B1	131.7 (2)	H10A—C10—H10C	109.5
N2—N1—B1	118.5 (2)	H10B—C10—H10C	109.5
C4—N2—N1	106.9 (2)	C12—C11—H11A	109.5
C4—N2—Co1	131.96 (19)	C12—C11—H11B	109.5
N1—N2—Co1	119.96 (17)	H11A—C11—H11B	109.5
C7—N3—N4	109.1 (2)	C12—C11—H11C	109.5
C7—N3—B1	132.0 (2)	H11A—C11—H11C	109.5
N4—N3—B1	118.2 (2)	H11B—C11—H11C	109.5
C9—N4—N3	107.4 (2)	N5—C12—C13	109.2 (2)
C9—N4—Co1	131.58 (18)	N5—C12—C11	120.9 (2)
N3—N4—Co1	120.24 (16)	C13—C12—C11	129.8 (2)
C12—N5—N6	107.6 (2)	C14—C13—C12	105.9 (2)
C12—N5—Co1	132.25 (18)	C14—C13—H13	127.0
N6—N5—Co1	119.92 (16)	C12—C13—H13	127.0
C14—N6—N5	108.8 (2)	N6—C14—C13	108.5 (2)
C14—N6—B2	131.8 (2)	N6—C14—C15	122.6 (2)
N5—N6—B2	118.7 (2)	C13—C14—C15	128.8 (2)
C17—N7—N8	109.3 (2)	C14—C15—H15A	109.5
C17—N7—B2	131.5 (2)	C14—C15—H15B	109.5
N8—N7—B2	118.8 (2)	H15A—C15—H15B	109.5
C19—N8—N7	106.9 (2)	C14—C15—H15C	109.5
C19—N8—Co1	132.37 (19)	H15A—C15—H15C	109.5
N7—N8—Co1	118.93 (17)	H15B—C15—H15C	109.5
C2—C1—H1A	109.5	C17—C16—H16A	109.5
C2—C1—H1B	109.5	C17—C16—H16B	109.5
H1A—C1—H1B	109.5	H16A—C16—H16B	109.5
C2—C1—H1C	109.5	C17—C16—H16C	109.5
H1A—C1—H1C	109.5	H16A—C16—H16C	109.5
H1B—C1—H1C	109.5	H16B—C16—H16C	109.5
N1—C2—C3	107.9 (2)	N7—C17—C18	108.4 (2)
N1—C2—C1	123.6 (3)	N7—C17—C16	123.1 (3)
C3—C2—C1	128.5 (3)	C18—C17—C16	128.5 (3)
C4—C3—C2	106.5 (2)	C17—C18—C19	106.0 (2)
C4—C3—H3	126.8	C17—C18—H18	127.0
C2—C3—H3	126.8	C19—C18—H18	127.0
N2—C4—C3	109.3 (2)	N8—C19—C18	109.3 (2)
N2—C4—C5	121.6 (3)	N8—C19—C20	121.3 (2)
C3—C4—C5	129.1 (3)	C18—C19—C20	129.4 (3)
C4—C5—H5A	109.5	C19—C20—H20A	109.5
C4—C5—H5B	109.5	C19—C20—H20B	109.5
H5A—C5—H5B	109.5	H20A—C20—H20B	109.5
C4—C5—H5C	109.5	C19—C20—H20C	109.5
H5A—C5—H5C	109.5	H20A—C20—H20C	109.5
H5B—C5—H5C	109.5	H20B—C20—H20C	109.5
C7—C6—H6A	109.5	N3—B1—N1	110.2 (2)
C7—C6—H6B	109.5	N3—B1—H1D	109.4
H6A—C6—H6B	109.5	N1—B1—H1D	109.6
C7—C6—H6C	109.5	N3—B1—H1E	109.7
H6A—C6—H6C	109.5	N1—B1—H1E	109.7
H6B—C6—H6C	109.5	H1D—B1—H1E	108.2
N3—C7—C8	108.3 (2)	N6—B2—N7	109.7 (2)
N3—C7—C6	123.0 (2)	N6—B2—H2A	109.7
C8—C7—C6	128.7 (2)	N7—B2—H2A	109.8
C7—C8—C9	106.0 (2)	N6—B2—H2B	109.6
C7—C8—H8	127.0	N7—B2—H2B	109.7
C9—C8—H8	127.0	H2A—B2—H2B	108.3
			
C2—N1—N2—C4	0.8 (3)	N1—N2—C4—C5	179.3 (2)
B1—N1—N2—C4	174.7 (2)	Co1—N2—C4—C5	11.9 (4)
C2—N1—N2—Co1	170.05 (17)	C2—C3—C4—N2	0.6 (3)
B1—N1—N2—Co1	−16.1 (3)	C2—C3—C4—C5	−179.6 (3)
N8—Co1—N2—C4	−90.4 (3)	N4—N3—C7—C8	0.1 (3)
N5—Co1—N2—C4	30.3 (3)	B1—N3—C7—C8	170.1 (3)
N4—Co1—N2—C4	144.3 (2)	N4—N3—C7—C6	−178.0 (2)
N8—Co1—N2—N1	103.6 (2)	B1—N3—C7—C6	−8.0 (4)
N5—Co1—N2—N1	−135.75 (18)	N3—C7—C8—C9	0.0 (3)
N4—Co1—N2—N1	−21.76 (19)	C6—C7—C8—C9	178.0 (3)
C7—N3—N4—C9	−0.2 (3)	N3—N4—C9—C8	0.2 (3)
B1—N3—N4—C9	−171.7 (2)	Co1—N4—C9—C8	169.59 (19)
C7—N3—N4—Co1	−171.03 (17)	N3—N4—C9—C10	179.1 (2)
B1—N3—N4—Co1	17.4 (3)	Co1—N4—C9—C10	−11.5 (4)
N2—Co1—N4—C9	−147.1 (2)	C7—C8—C9—N4	−0.1 (3)
N8—Co1—N4—C9	68.6 (3)	C7—C8—C9—C10	−178.9 (3)
N5—Co1—N4—C9	−36.9 (3)	N6—N5—C12—C13	−0.7 (3)
N2—Co1—N4—N3	21.2 (2)	Co1—N5—C12—C13	173.49 (19)
N8—Co1—N4—N3	−123.16 (19)	N6—N5—C12—C11	177.2 (2)
N5—Co1—N4—N3	131.42 (18)	Co1—N5—C12—C11	−8.6 (4)
N2—Co1—N5—C12	66.4 (3)	N5—C12—C13—C14	0.8 (3)
N8—Co1—N5—C12	−149.9 (2)	C11—C12—C13—C14	−176.8 (3)
N4—Co1—N5—C12	−37.8 (3)	N5—N6—C14—C13	0.2 (3)
N2—Co1—N5—N6	−119.98 (19)	B2—N6—C14—C13	169.9 (3)
N8—Co1—N5—N6	23.8 (2)	N5—N6—C14—C15	−177.4 (2)
N4—Co1—N5—N6	135.80 (18)	B2—N6—C14—C15	−7.7 (4)
C12—N5—N6—C14	0.3 (3)	C12—C13—C14—N6	−0.6 (3)
Co1—N5—N6—C14	−174.73 (17)	C12—C13—C14—C15	176.8 (3)
C12—N5—N6—B2	−170.9 (2)	N8—N7—C17—C18	−0.5 (3)
Co1—N5—N6—B2	14.0 (3)	B2—N7—C17—C18	−173.1 (3)
C17—N7—N8—C19	1.0 (3)	N8—N7—C17—C16	180.0 (2)
B2—N7—N8—C19	174.6 (2)	B2—N7—C17—C16	7.4 (5)
C17—N7—N8—Co1	167.53 (18)	N7—C17—C18—C19	−0.1 (3)
B2—N7—N8—Co1	−18.8 (3)	C16—C17—C18—C19	179.4 (3)
N2—Co1—N8—C19	−95.1 (3)	N7—N8—C19—C18	−1.1 (3)
N5—Co1—N8—C19	141.3 (2)	Co1—N8—C19—C18	−165.08 (19)
N4—Co1—N8—C19	26.8 (3)	N7—N8—C19—C20	177.5 (2)
N2—Co1—N8—N7	102.4 (2)	Co1—N8—C19—C20	13.5 (4)
N5—Co1—N8—N7	−21.16 (19)	C17—C18—C19—N8	0.8 (3)
N4—Co1—N8—N7	−135.73 (18)	C17—C18—C19—C20	−177.6 (3)
N2—N1—C2—C3	−0.5 (3)	C7—N3—B1—N1	127.9 (3)
B1—N1—C2—C3	−173.3 (3)	N4—N3—B1—N1	−62.9 (3)
N2—N1—C2—C1	179.7 (2)	C2—N1—B1—N3	−125.5 (3)
B1—N1—C2—C1	7.0 (4)	N2—N1—B1—N3	62.3 (3)
N1—C2—C3—C4	−0.1 (3)	C14—N6—B2—N7	130.1 (3)
C1—C2—C3—C4	179.7 (3)	N5—N6—B2—N7	−61.1 (3)
N1—N2—C4—C3	−0.9 (3)	C17—N7—B2—N6	−123.8 (3)
Co1—N2—C4—C3	−168.27 (19)	N8—N7—B2—N6	64.2 (3)
